# Effects of COVID-19 Pandemic on Working Conditions of Dentists in Poland and Turkey

**DOI:** 10.3390/medicina57101082

**Published:** 2021-10-11

**Authors:** Sarkis Sozkes, Iwona Olszewska-Czyż

**Affiliations:** 1 Biomedical Engineering Biomaterials Department, Tekirdag Namik Kemal University, Tekirdag 59860, Turkey; 2 Periodontology and Clinical Oral Pathology Department, Jagiellonian University Medical College, 31155 Krakow, Poland; iwona.olszewska-czyz@uj.edu.pl

**Keywords:** COVID-19, dentists, SARS-CoV-2, PPE personal protective equipment, occupational medicine, working conditions

## Abstract

*Background and Objectives*: Due to the specific working conditions dental professionals represent a group of high risk of infection and COVID-19 pandemic in many ways have influenced their working environment. The aim of this study was to evaluate effects of COVID-19 pandemic on working conditions of dentists in Poland and Turkey. *Materials and Methods*: The study was an anonymous online questionnaire conducted among thedentists in two countries: Poland and Turkey. The survey consisted of general questions, COVID-19 pandemic infection and working history as well as working conditions before and during pandemic. Chi-square test, Fisher’s Exact test, Fisher Freeman Halton test and Continuity (Yates) Correction were used to compare qualitative data. *Results*: The study was conducted with a total of 400 participants, 162 (40.5%) men and 238 (59.5%) women, aged between 23 and 67. The mean age of the participants was 42.39 ± 9.99 years. Positive COVID-19 test results among dentists in Poland were found to be significantly higher than in Turkey. Time of dental procedures during the COVID-19 pandemic in Poland and Turkey was significantly increased. The usage of N95/FFP2 or N99/FFP3 masks and surgical gowns during COVID-19 pandemic compared to pre-COVID-19 periods was clearly higher (*p* < 0.05). Reusable full-face and half-face elastomeric respirators are increasingly used in Turkey. During the COVID-19 pandemic a 25% decrease in dentists’ income in Poland (81%) was significantly high than in Turkey (47.5%). *Conclusions*: COVID-19 pandemic has influenced working conditions of dentists. Many dentists got infected during the pandemic, dental procedures’ time has increased, and protective equipment usage has become higher. Further studies analyzing the working conditions of dentists during COVID-19 pandemic should be conducted for better planning of future decisions taken by governments and authorities.

## 1. Introduction

The World Health Organization (WHO) declared the novel coronavirus disease 2019 (COVID-19) a pandemic on March 11, 2020 [1]. Globally, as of 14 July 2021, more than 186 million confirmed cases of COVID-19 and unfortunately more than 4 million deaths were reported by WHO. It was reported that more than 3.4 billion COVID-19 vaccine doses have been administered [2]. The year 2020 and first quarter of 2021 has passed under the influence of the COVID-19 pandemic, and there have been measures and restrictions affecting social life in many countries. Health services have focused on canceling or delaying many non-urgent treatments to give priority to individuals affected by the pandemic [3].

Oral and dental health treatments have an important place among health services [4]. The necessity of providing uninterrupted emergency dental treatment to patients is a reality accepted by everyone and primarily all non-urgent dental treatments were postponed. As a result of the prolongation of the pandemic process, dental problems that were not very urgent, but likely to cause permanent damage in case of delay, have to be followed now and cannot be postponed anymore. The most frequent dental emergency cases are: tooth fractures, pulpal infections, periodontal abscesses, fractures of prosthetic restorations and urgent extractions, however there are many other conditions within the oral cavity which, even though not urgent, may result in serious oral and also general health issues [5].

Due to the nature of their working conditions, dentists communicate very closely with virus carrier patients, are exposed to saliva, blood and other body fluids, and therefore face the risk of 2019-nCoV infection. During the COVID-19 pandemic, many authorities have published guidelines on the precautions to be taken against COVID-19 during the implementation of oral and dental health services and the provision of treatment services. Infection control measures are necessary to prevent further spread of the virus and to help control the pandemic situation. Dental treatments should be carried out in protective and preventive environment. Dentists should protect themselves by using personal equipment and also protect their patients from contamination by taking pandemic measures [6,7,8,9]. These pandemic measures require a higher usage of protective equipment what not only increase the expenses but also consume more chair time for dental procedures. During pandemic governments also ruled lockdowns and limitations of public mobility. Apart from all those factors, dentists were also afraid to work because of the fear of being infected.

Considering all above aspects, a hypothesis of influence of COVID-19 pandemic on working conditions of dentists has been raised parallel to possible differences between countries and other factors related to dental environment nowadays.

### 1.1. Objectives

The aim of the study was to evaluate effects of COVID-19 pandemic on working conditions of Polish and Turkish dentists and to investigate any differentiating factors relevantly. The main research objective was to investigate if the COVID-19 pandemic changed the working conditions of the dentists and analyze eventual differences between nationalities and different aspects affecting the dentists during pandemic.

### 1.2. Trial Design

The study was an anonymous online questionnaire based trial conducted among dentists in two countries: Poland and Turkey. It was performed in accordance with Public Opinion Research Guidelines and based on the Computer Assisted Web Interview methodology. Ethical approval for this study was obtained.

## 2. Materials and Methods

### 2.1. Participants

The study was conducted among 400 dentists: 200 in Poland and 200 in Turkey. Only the dentists working in Poland or in Turkey were enrolled in the study. Participation was voluntary and participants were allowed to terminate the survey at any time. Confidentiality and privacy was protected according to General Data Protection Regulation.

### 2.2. Data Collection

Online social media Facebook and Twitter were employed for sampling. The survey was posted in the form of a link to be filled in directly by a person willing to participate. The questionnaire was placed in professional social media groups (in which members are subject to prior verification confirming the dental license) and only to groups which allow posting content of this type.

### 2.3. Questionnaire

The authors followed Study Protocol for an Online Questionnaire Survey to build up and develop the questionnaire [10]. The survey consisted of two parts. First part comprised of demographic data, including age, gender, country of origin, COVID -19 infection history (being tested for COVID-19, test results, hospitalization, symptoms), place of work (private, public, mixed), lockdown history, working status during pandemic (changes in treatment time and income) and opinion about the risk of COVID-19 infection. The dentists were also asked to self-assess their perception of having enough knowledge about Covid-19. Second part of the survey consisted of questions related to the usage of protective equipment and prophylactic procedures. The dentists were asked to mark what they have used before pandemic and what they are using during it out from: masks (surgical, N95, N99/FFP2, FFP3), reusable full-face respirators, reusable half-face respirators, gowns (sterile disposable, disposable), glasses(protection), gloves (sterile, non-sterile), anti-retraction system valve rotaries, triage and disinfection procedures. A pre-testing of the questionnaire was carried out with 20 dentists. Then Interclass Correlation Coefficients for Reliability was controlled and discussed to create the final structure of the questionnaire [11,12]. To evaluate the study parameters age, gender, country of residence and working status questions were included in the questionnaire.

### 2.4. Statistical Methods

IBM SPSS Statistics 22 (IBM SPSS, Istanbul, Turkey) program was used for statistical analysis. The conformity of the parameters to the normal distribution was evaluated with the Shapiro Wilks test. Student’s *t* test was used for the comparison of normally distributed parameters (quantitative data) between two groups in addition to descriptive statistical methods (mean, standard deviation, frequency). Chi-square test, Fisher’s Exact test, Fisher Freeman Halton test and Continuity (Yates) Correction were used to compare qualitative data. Significance was evaluated at the *p* < 0.05 level.

### 2.5. Ethics

The study was performed in accordance with the Helsinki Declaration and official approval from the Jagiellonian University Ethics Committee was obtained (No.1072.6120.157.2021).

## 3. Results

The study was conducted among 400 participants, 162 (40.5%) men and 238 (59.5%) women, aged between 23 and 67, between 9–12 July 2021. The mean age of the participants was 42.39 ± 9.99 years. The study was conducted in 2 countries: 200 (50%) Poland and 200 (50%) Turkey.

Evaluation of study parameters across countries are listed in Table 1. Employment in the private sector in Poland was found to be statistically significantly lower than in Turkey. The rate of positive COVID-19 test results in Poland (51%) was found to be higher than Turkey (16%), and the hospitalization rate due to COVID-19 in Poland (6%) was found to be higher than in Turkey (2%). The symptoms development rate (52%) in Poland due to COVID-19 was found to be statistically significantly higher than in Turkey (15%).

Working history of participants during COVID-19 showed that one-fourth of dentists (23%) mostly from Turkey has continued to work as usual, whereas approximately two-thirds of dentists (63.7%): 162 Polish and 93 Turkish dentists have had a break (lock down) to their practice for some period. Even though there were significant differences in percentage of dentists who kept working as usual and the ones having breaks in dental services by stopping all activities for some periods (due to lockdowns, quarantines, COVID-19 infection etc.), the emergencies were treated in both countries by similar number of dentists, usually the ones who limited their service to urgent procedures.

Treatment time for dental procedures during the COVID-19 pandemic in Poland was found to be 25% longer and significantly higher than in Turkey, whereas decrease in income among Polish dentists (81%) was found to be statistically higher than among Turkish dentists (47.5%).

Polish dentists self assessed their perception of having enough knowledge about COVID-19 significantly lower than dentists in Turkey. Meanwhile the rate of Polish dentists marking that COVID-19 is most likely a risk for infection for them; being unsure of ability to prevent the transmission of COVID-19 during business activities and apprehension of infection transmission’s risk in the dental practice was found to be higher than in Turkey.

The participants, who have mentioned that they had COVID-19 positive test results, were also asked to give the details about their symptoms (Table 2).

The rate of symptoms such as fever, cough, fatigue, pain and headache was significantly higher in Poland than in Turkey. This correlates with higher number of positive COVID-19 test results and symptoms development rate among Polish dentists in comparison to Turkish ones.

Evaluation of equipment used in dental clinics before COVID-19 between countries Poland and Turkey mostly gave similar results before COVID-19 (Table 3).

Evaluation of equipment used during COVID-19 in Poland and Turkey mostly gave similar results (Table 4). The rates of usage of surgical masks and N95/FFP2 or N99/FFP3 masks in Poland and Turkey were significantly high. Reusable full face mask and half face mask usage rate was significantly higher in Turkey (23%; 16.5%). All survey participants in Poland prefer to use disposable gowns (100%) during the COVID-19 pandemic, whereas in Turkey even though most of participants prefer to use disposable gowns (65%) many other also use sterile disposable gowns (32.5%).

## 4. Discussion

There are not many researches in the subject of the working environment and conditions of dentists during the COVID-19 pandemic. Most of the existing research have been carried out in the form of online surveys due to the pandemic restrictions in many countries. In the cross-sectional study performed in Switzerland and Liechtenstein, the economic effects of the protective measures taken by the governments were examined [8]. The authors pointed out that the strategies for additional protective measures should be better defined and that political decision makers should consider the serious economic implications when creating new rules. It has been reported that drastic measures such as “isolation”, which can lead to closures and unemployment, should be taken into account labor and economic losses.

Another published article evaluated COVID-19 preventive measures: awareness and perception among Italian dentists in Lombardy [9]. In this survey study, symptoms were collected in regions where the prevalence of the disease was different. Although this survey reported that dentists in the region where COVID-19 was most prevalent, had more symptoms than the rest of the sample, those who adopted a few precautionary measures were more confident in avoiding infection.

In our study the number of positive COVID-19 test results among dentists in Poland was found to be significantly higher than in Turkey, what consequently might explain the three times higher hospitalization rate in investigated group in Poland. The symptoms development rate in Poland due to COVID-19 was found to be statistically significantly higher than in Turkey, and the symptoms were: fever, cough, fatigue, pain and headache as also reported by Cagetti et al. [9].

Dental-related aspects on COVID-19 pandemic were also studied by another recent survey conducted on 440 participants from Central Italy [13]. Results of this research revealed that most professionals respected the advices given by authorities, showing parallel results with our study. In Central Italy mainly only emergency procedures were performed during the lockdown and the dental pulp inflammation treatment was one of the most frequent procedures. Authors concluded with very important recommendations stating that procedures such as telemedicine and triage are useful tools to assess patients conditions before the dental visit [13].

The oral manifestation of SARS-CoV-2 and the importance of the professional figure of the dentist in the diagnosis of COVID-19 should be discussed as well. In an observational human study on 20 patients hospitalized in Chieti, Italy, such oral manifestation as xerostomia and low salivary flow rate has been followed, even though according to the evidence, it was hard to conclude that those clinical conditions were due to SARS-CoV-2 infection [14]. Furthermore, the dysgeusia symptom was reported as a warning signal for the patients. The decrease of the proper oral hygiene level among hospitalized patients was also noticed. Authors emphasized the importance of the dentists in following the infected patients and suggested to have a team specialized in dentistry within hospitals treating the COVID-19 patients [14].

Treatment time of dental procedures during the COVID-19 pandemic in Poland and Turkey was found to be increased. This has to be carefully evaluated considering the workforce of dentistry. As also mentioned in article published by Wolf et al. the lockdowns have important effect on income of dentists [8]. In our survey we found that Polish dentists had more decrease in their income, which may be explained by lockdowns. In Turkey decrease in income may have been felt less than in Poland. Dentists in public service were directed to COVID-19 testing and vaccination activities by Ministry of Health, what nearly caused the public dental clinics stopping dental services and parallel more patients were treated in private clinics.

Evaluation of the changes in equipment usage before and during COVID-19 pandemic in Poland reveals the increase in the usage of N95/FFP2 or N99/FFP3 masks during pandemic compared to pre-COVID-19 periods. Also the usage of surgical gown was statistically significant. It may be very easily explained by the working protection guidelines for Polish dentists created by authorities for the pandemic period. The decrease in normal surgical masks’ usage in Turkey may be caused by high increase in the use of more protective masks such as N95/FFP2 or N99/FFP3 during pandemic compared to pre-COVID-19 time [6]. Reusable full-face and half-face elastomeric respirators were also increasingly used in Turkey during COVID-19.

Lack of uniform blueprints and guidelines for working conditions of dentists during the pandemic such as the COVID-19 was also mentioned by Wiesmüller et al. [13]. The high risk of infection in the dental working conditions in Austria, Germany and Switzerland was acknowledged in this study as well as the need of evaluation of the number of infections among dentists in other European countries was mentioned. Also previously mentioned studies suggest more researches on the influence of pandemic on dental working environment should be carried out [8,9,15]. Thus our study reports data from two countries in Europe and allows making primary conclusions on how the working conditions of the dentists were affected by COVID-19.

## 5. Conclusions

The increase in the dental treatment procedures’ time, higher usage of personal protective equipment, bigger risk of infection at work and decrease in income during COVID-19 pandemic were expected results of our study. Even though all efforts were given to reach as many dentists as possible, the study investigates random group of 400 dentists, but despite this limitation of this research, there were enough data for primary analysis. Regardless the fact that the evaluation was addressed to dentists from two countries and pandemic situation may vary from country to country in terms of infection rate, legislation etc., when the specific dental working conditions and evidence is taken into account, the overall conclusions may be addressed in a wider perspective. Many different aspects of the evaluated dental working conditions were affected by COVID-19 pandemic in both countries. The study results reveal the increase in the dental treatment procedures’ time compared to pre-pandemic situation in Turkey and Poland. Also the usage of protective equipment was higher, what can be considered as an additional cost for dentists. At the same time there was a decrease of income reported in as well as many dentists got infected at work in Turkey and Poland.

Governments, national bodies and all those who influence the workplaces of dentists have to introduce minimum standards for facilities and working conditions for dentists during pandemic. Countries should also significantly increase their investments in the healthcare sector in order to improve the working conditions and occupational safety of dentists. Further studies in more countries or multi-centered international researches analyzing the working conditions of dentists during COVID-19 pandemic would be a very important source for regulating institutions to prepare guidelines for similar future pandemic situations.

## Figures and Tables

**Table 1:** Evaluation of study parameters across countries.

		Country		Total	*p*
		Poland	Turkey		
		M ± SD	M ± SD	M ± SD	
Age		41.32 ± 8.88	43.46 ± 10.91	42.39 ± 9.99	0.032 ^1,^*
		***n* (%)**	***n* (%)**	***n* (%)**	
Gender	Male	74 (37%)	88 (44%)	162 (40.5%)	0.154 ^2^
	Female	126 (63%)	112 (56%)	238 (59.5%)	
Working status	Private	83 (41.5%)	127 (63.5%)	210 (52.5%)	0.000 ^2,^*
	Public	51 (25.5%)	38 (19%)	89 (22.3%)	
	Mixed	66 (33%)	35 (17.5%)	101 (25.3%)	
Positive COVID-19 test result	Yes	108 (54%)	32 (16%)	140 (35%)	0.000 ^2,^*
	No	10 (5%)	168 (84%)	178 (44.5%)	
	No test	82 (41%)	0 (0%)	82 (20.5%)	
Hospitalization rate due to COVID-19	Yes	12 (6%)	4 (2%)	16 (4%)	0.000 ^2,^*
	No	98 (49%)	28 (14%)	126 (31.5%)	
	NA	90 (45%)	168 (84%)	258 (64.5%)	
Symptoms development rate	Yes	104 (52%)	30 (15%)	134 (33.5%)	0.000 ^3,^*
	No	4 (2%)	2 (1%)	6 (1.5%)	
	NA	92 (46%)	168 (84%)	260 (65%)	
Currently working	Yes	200 (100%)	181 (90.5%)	381 (95.3%)	0.000 ^4,^*
	No	0 (0%)	19 (9.5%)	19 (4.8%)	
COVID-19 working history	As Usual	14 (7%)	78 (39%)	92 (23%)	0.000 ^2,^*
	Emergencies	24 (12%)	29 (14.5%)	53 (13.3%)	
	Break	162 (81%)	93 (46.5%)	255 (63.7%)	
Treatment time for dental procedures	<25%	0 (0%)	16 (8%)	16 (4%)	0.000 ^2,^*
	>25%	200 (100%)	120 (60%)	320 (80%)	
	No change	0 (0%)	64 (32%)	64 (16%)	
Income of dentists	D < 25%	162 (81%)	95 (47.5%)	257 (64.3%)	0.000 ^2,^*
	D > 25%	24 (12%)	36 (18%)	60 (15%)	
	No change	14 (7%)	69 (34.5%)	83 (20.8%)	
COVID-19 self-assessed perception of knowledge	Yes	0 (0%)	145 (72.5%)	145 (36.3%)	0.000 ^4,^*
	No	200 (100%)	55 (27.5%)	255 (63.7%)	
Risk of infection for the dentist	Very likely	200 (100%)	141 (70.5%)	341 (85.3%)	0.000 ^2,^*
	Likely	0 (0%)	49 (24.5%)	49 (12.3%)	
	Unlikely	0 (0%)	10 (5%)	10 (2.5%)	
Infection avoidance at clinic	NC	200 (100%)	44 (22%)	244 (61%)	0.000 ^2,^*
	AC	0 (0%)	56 (28%)	56 (14%)	
	C	0 (0%)	100 (50%)	100 (25%)	
Rate of apprehension of infection transmission’s risk	Higher	200 (100%)	87 (43.5%)	287 (71.8%)	0.000 ^2,^*
	Lower	0 (0%)	76 (38%)	76 (19%)	
	Same	0 (0%)	37 (18.5%)	37 (9.3%)	

^1^ Student t Test. ^2^ Ki-Kare Test. ^3^ Fisher Freeman Halton Test. ^4^ Continuity (Yates) Correction. * *p* < 0.05. Notes: NA: Not applicable, NC: No confident, AC: Abit confident, C: Confident, M ± SD: Mean ± Standart deviation.

**Table 2:** Evaluation of COVID-19 symptoms across countries.

You Had One/More Symptoms/Signs		Country		Total	*p*
		Poland	Turkey		
		*n* (%)	*n* (%)	*n* (%)	
Fever	Yes	86 (43%)	14 (7%)	100 (25%)	0.000 ^1,^*
	No	22 (11%)	18 (9%)	40 (10%)	
	NA	92 (46%)	168 (84%)	260 (65%)	
Cough	Yes	68 (34%)	9 (4.5%)	77 (19.3%)	0.000 ^1,^*
	No	40 (20%)	23 (11.5%)	63 (15.8%)	
	NA	92 (46%)	168 (84)%	260 (65%)	
Fattigue	Yes	86 (43%)	25 (12.5%)	111 (27.8%)	0.000 ^1,^*
	No	22 (11%)	7 (3.5%)	29 (7.2%)	
	NA	92 (46%)	168 (84%)	260 (65%)	
Pain	Yes	86 (43%)	9 (4.5%)	95 (23.8%)	0.000 ^1,^*
	No	22 (11%)	23 (11.5%)	45 (11.3%)	
	NA	92 (46%)	168 (84%)	260 (65%)	
Headache	Yes	81 (40.5%)	16 (8%)	97 (24.3%)	0.000 ^1,^*
	No	27 (13.5%)	16 (8%)	43 (10.8%)	
	NA	92 (46%)	168 (84%)	260 (65%)	

^1^ Ki-Kare Test. * *p* < 0.05. Note: NA: Not applicable.

**Table 3:** Evaluation of equipment used before COVID-19 in both countries.

Which of the Following Protective Equipment Did You Wear/Use before COVID-19 Pandemic?		Country		Total	*p*
		Poland	Turkey		
		*n* (%)	*n* (%)	*n* (%)	
Masks (Surgical)	Yes	200 (100%)	187 (93.5%)	387 (96.8%)	0.001 ^1,^*
	No	0 (0%)	13 (6.5%)	13 (3.3%)	
Masks (N95,N99/FFP2,FFP3)	Yes	0 (0%)	38 (19%)	38 (9.5%)	0.000 ^1,^*
	No	200 (100%)	162 (81%)	362 (90.5%)	
Reusable Full-face Respirators	Yes	0 (0%)	11 (5.5%)	11 (2.8%)	0.002 ^1,^*
	No	200 (100%)	189 (94.5%)	389 (97.3%)	
Reusable Half-face Respirators	Yes	0 (0%)	12 (6%)	12 (3%)	0.001 ^1,^*
	No	200 (100%)	188 (94%)	388 (97%)	
Gown (Sterile disposable)	Yes	0 (0%)	29 (14.5%)	29 (7.2%)	0.000 ^1,^*
	No	200 (100%)	171 (85.5%)	371 (92.8%)	
Gown (Disposable)	Yes	0 (0%)	96 (48%)	96 (24%)	0.000 ^1,^*
	No	200 (100%)	104 (52%)	304 (76%)	
Glasses(protection)	Yes	200 (100%)	120 (60%)	320 (80%)	0.000 ^1,^*
	No	0 (0%)	80 (40%)	80 (20%)	
Gloves Sterile	Yes	200 (100%)	91 (45.5%)	291 (72.8%)	0.000 ^1,^*
	No	0 (0%)	109 (54.5%)	109 (27.3%)	
Gloves Non-sterile	Yes	200 (100%)	172 (86%)	372 (93%)	0.000 ^1,^*
	No	0 (0%)	28 (14%)	28 (7%)	
Anti-retraction system valve rotaries	Yes	200 (100%)	0 (0%)	200 (50%)	0.000 ^1,^*
	No	0 (0%)	200 (100%)	200 (50%)	

^1^ Continuity (Yates) Correction. * *p* < 0.05.

**Table 4:** Evaluation of equipment used during the COVID-19 pandemic in both countries.

Which of the Following Protective Equipment Did You Wear/Use during COVID-19 Pandemic?		Country		Total	*p*
		Poland	Turkey		
		*n* (%)	*n* (%)	*n* (%)	
Masks (Surgical)	Yes	200 (100%)	162 (81%)	362 (90.5%)	0.000 *
	No	0 (0%)	38 (19%)	38 (9.5%)	
Masks (N95,N99/FFP2,FFP3)	Yes	200 (100%)	183 (91.5%)	383 (95.8%)	0.000 *
	No	0 (0%)	17 (8.5%)	17 (4.3%)	
Reusable Full-face Respirators	Yes	0 (0%)	33 (16.5%)	33 (8.3%)	0.000 *
	No	200 (100%)	167 (83.5%)	367 (91.8%)	
Reusable Half-face Respirators	Yes	0 (0%)	46 (23%)	46 (11.5%)	0.000 *
	No	200 (100%)	154 (77%)	354 (88.5%)	
Gown (Sterile disposable)	Yes	0 (0%)	65 (32.5%)	65 (16.3%)	0.000 *
	No	200 (100%)	135 (67.5%)	335 (83.8%)	
Gown (Disposable)	Yes	200 (100%)	130 (65%)	330 (82.5%)	0.000 *
	No	0 (0%)	70 (35%)	70 (17.5%)	
Glasses(protection)	Yes	200 (100%)	161 (80.5%)	361 (90.3%)	0.000 *
	No	0 (0%)	39 (19.5%)	39 (9.8%)	
Gloves Sterile	Yes	200 (100%)	91 (45.5%)	291 (72.8%)	0.000 *
	No	0 (0%)	109 (54.5%)	109 (27.3%)	
Gloves Non-sterile	Yes	200 (100%)	172 (86%)	372 (93%)	0.000 *
	No	0 (0%)	28 (14%)	28 (7%)	
Anti-retraction system valve rotaries	Yes	200 (100%)	27 (13.5%)	227 (56.8%)	0.000 *
	No	0 (0%)	173 (86.5%)	173 (43.3%)	

Continuity (Yates) Correction. * *p* < 0.05.

## Data Availability

The data presented in this study are available on request from the corresponding author. The data are not publicly available due to ethical restrictions.
